# How safe are children with COVID-19 from cardiac risks? Pediatric risk assesment; insights from echocardiography and electrocardiography

**DOI:** 10.3906/sag-2010-240

**Published:** 2021-06-28

**Authors:** Berna Şaylan ÇEVİK, Şule ARICI, Zeynep ERGENÇ, Eda KEPENEKLİ, Özge GÜNAL, Nurhayat YAKUT

**Affiliations:** 1 Department of Pediatric Cardiology, Marmara University School of Medicine, İstanbul Turkey; 2 Department of Pediatric Infection Disease, Marmara University School of Medicine, İstanbul Turkey; 3 Department of Pediatrics, Marmara University School of Medicine, İstanbul Turkey

**Keywords:** Children, COVID-19, electrocardiography, risk

## Abstract

**Background/aim:**

Approximately 40 million individuals worldwide have been infected with SARS-CoV-2, the virus responsible for the novel coronavirus disease-2019 (COVID-19). Despite the current literature about the cardiac effects of COVID-19 in children, more information is required. We aimed to determine both cardiovascular and arrhythmia assessment via electrocardiographic and echocardiographic parameters.

**Materials and methods:**

We evaluated seventy children who were hospitalized with COVID-19 infections and seventy children as normal control group through laboratory findings, electrocardiography (ECG), and transthoracic echocardiography (TTE).

**Results:**

We observed significantly increased levels of Tp-Te, Tp-Te/QT, and Tp-Te/QTc compared with the control group. Twenty-five of 70 (35.7%) patients had fragmented QRS (fQRS) without increased troponin levels. On the other hand, none of the patients had pathologic corrected QT(QTc) prolongation during the illness or its treatment. On TTE, 20 patients had mild mitral insufficiency, among whom only five had systolic dysfunction (ejection fraction < 55%). There was no significant difference between the patient and control groups, except for isovolumic relaxation time (IVRT) in terms of mean systolic and diastolic function parameters. IVRT of COVID patients was significantly lower than that of control group.

**Conclusion:**

Despite all the adult studies, the effects of COVID‐19 on myocardial function are not well established in children. The thought that children are less affected by the illness may be a misconception.

## 1. Introduction

The coronavirus disease (COVID-19), which started in Wuhan, China, in December 2019, and was declared a worldwide pandemic by the World Health Organization (WHO) on March 11th, 2020, is a novel infectious disease that causes respiratory illness and death [1]. Millions of individuals worldwide have been infected by COVID-19, with approximately 40 million infections worldwide at the time of writingWorld Health Organization. Coronavirus disease 2019 (COVID‐19): situation report– 70.2020. https://www.who.int/docs/defaultsource/coronavirus/situationreports/20200330‐sitrep‐70‐covid‐19.pdfsfvrsn=7e0fe3f8-4. [Accessed 30 March 2020]. . Most of the studies in the literature about the cardiac manifestations of COVID-19 including myocarditis and arrhythmias, electrocardiographic changes including ST segment-T wave changes, and laboratory abnormalities such as elevated high-sensitivity troponin T and N-terminal pro-brain-type natriuretic peptide (NT-pro BNP), have been in adults [2–4]. Such studies were also reported with fragmented QRS (fQRS). fQRS as a manifestation of local conduction abnormalities in the ventricular myocardium and scar tissue has been established several times in the literature [5].

COVID-19 can lead to cardiac injury via the following possible mechanisms [6]; 1) increased cytokines and immune-inflammatory response cause indirect injury, 2) direct invasion of cardiomyocytes with virus, 3) effects due to lung failure, and 4) hypoxia leading to oxidative stress and injury to cardiomyocytes. The pathophysiology is still unclear; why are these abnormalities related to increased mortality and morbidity during illness? 

In the first stages of the pandemic, COVID-19 was observed to cause lesser effects in children in clinical evaluation, but later, and with mutations of the virus, different and more serious signs and symptoms of the illness were seen in children by physicians [7].

In this study, we evaluated the cardiac functions of COVID-19 infected children who were hospitalized in a tertiary hospital using electrocardiography, echocardiography, and laboratory findings. 

## 2. Materials and methods

We presented a cross-sectional analysis of 70 children hospitalized with COVID-19 infections, who had respiratory distress, intractable fever, angina, and gastrointestinal symptoms in the first months of the disease (March 15th-June 30th 2020). 

The study was approved by the local ethics committee of University (Date: 24.07.2020-09.2020.717), and the Turkish Ministry of Health (2020-06-08/T19-55-10).

The patients were appraised through laboratory findings, electrocardiography (ECG), and transthoracic echocardiography (TTE). The patients were initially diagnosed according to clinical manifestations and exposure history. All patients were evaluated using laboratory markers such as white blood cell (WBC) count, C-reactive protein, Pro BNP, COVID-19, polymerase chain reaction (PCR), troponin T levels, coagulation parameters, chest X-ray, ECG, and TTE. Chest X-Rays were evaluated by two radiologist, who defined the results as normal, lober pneumonia, and ground-glass opacities were observed, which are the most common findings of the chest X-Rays of this disease [8].

On the ECG, the risk of arrhythmia was observed by the evaluation of depolarization and repolarization parameters (such as Tp-Te interval, Tp-Te/QTc, Tp-Te/QT ratio) and cardiac ventricle systolic and diastolic functions employing ejection fraction, fractional shortening, and Doppler parameters. In the acute phase of the illness, the ECG and TTE parameters were correlated with the normal age and sex-matched healthy children as control group (n = 70). 

### 2.1. Electrocardiographic measurements

**Figure 1:**
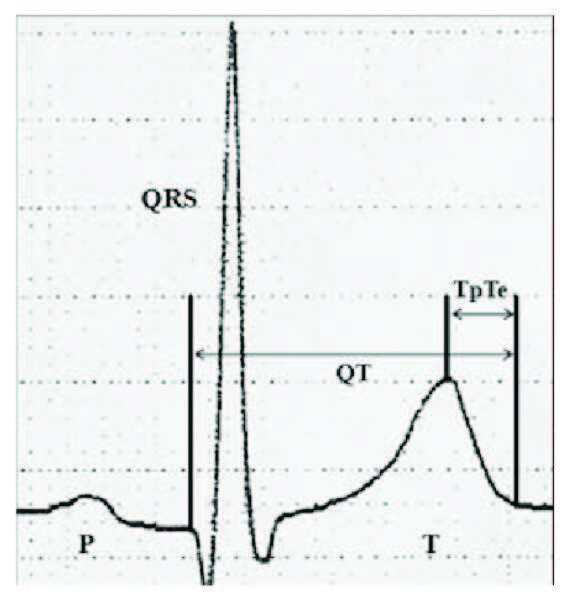
To calculate Tp-Te, Tp-Te/QT-, and QTc interval.

A 12-lead electrocardiogram with standard chest and limb leads was used to evaluate the Tp-Te interval, QTc interval, QTd interval, and presence of fQRS. The 12-lead ECG was recorded at a paper speed of 50 mm/s in the supine position. To decrease measurement errors, all ECGs were scanned and transferred to a personal computer and then used for 400% magnification with the Adobe Photoshop software. QTd was calculated as the difference between the longest and the shortest QT intervals. ECG measurements of QTc and Tp-Te intervals were performed by two cardiologists who were blinded to the patient data. Subjects with U waves on their ECGs were excluded from the study. An average value of 3 readings were calculated for each lead. The QT interval was measured from the beginning of the QRS complex to the end of the T wave and corrected for heart rate using the Bazett formula: cQT = QTÖ (RR interval). The Tp-Te interval was defined as the interval from the peak of the T wave to the end of the T wave. Measurements of the Tp-Te interval were performed from the precordial leads. The Tp-Te/QT ratio was calculated from these measurements [9] (Figure 1). Fragmentation of QRS was defined as the presence of various RSR’ patterns with different morphologies of QRS complexes. Various RSR’ patterns included additional R wave (R’), notching of the R wave or the S wave, or the presence of >1 R’ (fragmentation) without a typical bundle branch block in two contiguous leads corresponding to a major lead set for major coronary artery territory [10]. Any QRS morphology with a QRS duration >120 ms, including bundle branch block or intraventricular conduction delay, was excluded. 

### 2.2. Echocardiographic measurements

All patients underwent an echocardiographic examination using a Philips IE33-model echocardiography device (Phillips Medical Systems Andover, MD, USA) equipped with 5-MHz sector transducers. Two-dimensional echocardiographic images were obtained using apical four-chamber (4C) and (2C) views, and a parasternal long axis (LAX) view. Coronary artery involvement was also examined [11]. Echocardiography was also evaluated by two pediatric cardiologist (B.S.C;S.A). There was agreement between intraobserver and interobserver variability. 

**Left ventricular functions:**

Left ventricular dimensions, interventricular septal diameter (IVSD), LVPWD (left ventricular posterior wall diameter), and LVED (left ventricle end diastole) and LVEDS (left ventricle end systole) dimensions were measured, and left ventricular ejection and shortening fractions (LVEF-SF) were calculated using the Teichholtz formula [12]. Left ventricular diastolic functions were studied using pulse wave echocardiography with mitral inflow Doppler velocities; early (E wave) and late (A wave) velocities, deceleration time, and IVRT were measured. Isovolumic relaxation time (IVRT) was measured with PW Doppler, which the transducer is angulated into the apical 5-chamber or long-axis view and the sample volume placed within the left ventricle outflow tract (LVOT), but in proximity to the anterior mitral valve (MV) leaflet to record both inflow and outflow signals. Three cardiac cycles should be averaged when measuring transmitral velocities and IVRT.

## 3. Statistics

Statistical analysis was performed using the SPSS Statistics for Windows v.20 software package (SPSS, Inc., Chicago, IL, USA). The independent-sample t-test or Mann–Whitney U test was used to compare continuous variables. For categorical variables, the Chi-square test was used. Correlations were evaluated using univariate and multivariate linear regression analysis. A p-value of <0.05 was accepted as statistical significance. Inter and intra observer agreement was assessed as 95, 96% respectively.

## 4. Results

Demographic variables are given in Table 1. The patients had moderate-severe disease, as previously described in various studies [13]. Seventy patients were evaluated (girls 47.1%, boys 52.9%). The mean age of the patients was 8.3 ± 6.8 (minimum 1 month, maximum 18 years). None of the patients had an anamnesis of overseas contact, 65% had exposure to a patient 5–15 days before disease onset. The COVID-19 PCR test was negative in 18.6% of patients and positive in 81.4%. 

**Table 1:** Demographic characteristics of the patient and control groups.

	Patients(n = 70)	Controls(n = 70)	p
Mean age (years)	8.3 ± 6.8	8.32 ± 6.6	0.20
Sex	female 47.1%male 52.9%	female 50%male 50%	0.84
Weight (kg)	22.5 ± 5.6	23 ± 6.1	0.77
Height (cm)	126 ± 4.2	125 ± 5.4	0.70
BMI (kg/m2)	15.8 ± 1.1	15.6 ± 1.2	0.69
Overseas contact	none		
Exposure to virus	65%		
COVID-19 PCR	Positive 81.4%Negative 18.6%		

Covid: coronovirus disease, PCR: polymerase chain reaction, BMI: Body mass index.

Patients whose tests were negative were considered highly possible to have COVID-19 infection because of the clinical stature, laboratory markers, and the anamnesis of exposure to the virus. All these patients had direct exposure to the virus. 

**Figure 2:**
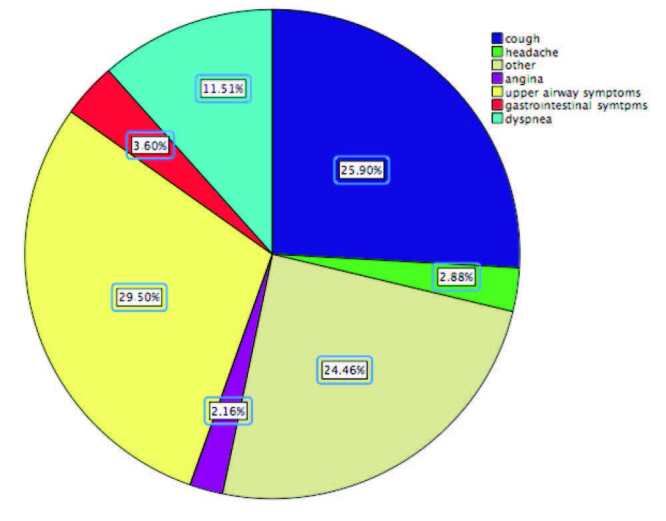
Symptom distributions of the children with COVID-19.

Of the patients admitted to hospital, 52.9% had a fever (body temperature range, 36.1–38.5 °C); 60% had a cough, 70% patients had symptoms of upper airway disease (nasal congestion and rhinorrhea), 30% had dyspnea, 14.3% had a headache, 8.8% had gastrointestinal system symptoms, 4.3% had angina, and 1.4% had anosmia. Some patients had two or three symptoms together (Figure 2).

The hematologic, serologic, and biochemical parameters are presented in Table 2. Increased levels of CRP and procalcitonin were detected. Troponin T, CK and CK-MB, and Pro BNP levels are shown in Table 3.

**Table 2:** Hematologic, serologic, and biochemical parameters of the patients.

CRP (mg/dL)	31.64 ± 47.81
Procalcitonin (mg/L)	0.63 ± 1.29
Hb (g/dL)	11.84 ± 2.19
Hct (%)	34 ± 6.25
WBC (/mm3)	7176 ± 2078
Neu (%)	51.2 ± 19
Plt (/mm3)	247.00 ± 133.00
BUN (mg/dL)	9.8 ± 5.7
Cr (mg/dL)	0.56 ± 0.38
AST(IU/L)	38 ± 32
ALT(IU/L)	22.83 ± 21

CRP: C-Reactive protein, Hb: Hemoglobin, Hct: Hematocrite, Wbc: white blood cell, Neu: neutrofile, Plt: platelet, BUN: blood urea nitrogen, Cr: creatine, AST: aspartate aminotransferase, ALT: alanine aminotransferase.

**Table 3:** Cardiac markers of the patients.

CK (U/L)	212.19 ± 57.9
CK-MB (pg/L)	2.18 ± 1.14
Troponin-T (ng/L)	17 ± 4.8
NT-Pro BNP (ng/L)	300

CK: Creatine kinase, CK-MB: Creatine kinase muscle brain NT Pro BNP: N terminal pro Brain natriuretic peptide.

Standard signs of the disease in thorax computed tomography (CT) were seen in 37.1% of the patients. Chest X-ray results were grouped by two different radiologists as normal (32.9%), lobar pneumonia (44.3%), and ground-glass opacities (22.8%). 

All patients were clinically evaluated using a standard cardiac examination, regardless of the disease severity. The heart rate was 111 ± 20.3/min (min: 79, max: 170/min). The mean systolic blood pressure was 106.53 ± 15.77 mm Hg, and the mean diastolic blood pressure was 68.63 ± 9.9 mm Hg. Oxygen saturation was 82–100 (mean: 96.70 ± 3.05) mm Hg.

On the ECG, we evaluated depolarization and repolarization parameters using QRS, QTd, QTc, Tp-Te intervals, Tp-Te/QTc, Tp-Te/QTd, fragmented QRS (fQRS), and pathologic ST-T changes. ECG parameters were presented in Table 4. Around 47.1% of the patients had fQRS. None of the patients had either arrhythmia or pathologic ST-T changes. 

**Table 4:** ECG parameters of the patient and control groups.

ECG parameters	Patients(n = 70)	Controls(n = 70)	p
PR (msn)	100.51 ± 17.47	66.28 ± 7.6	0.05
QRS (msn)	67.85 ± 11.65	56.10 ± 4.98	0.01
QTc (msn)	422 ± 51.3	389.42 ± 14.92	0.01
QTd (msn)	96.98 ± 33.95	83.42 ± 7.85	0.02
QTdc (msn)	80.81 ± 7.9	77.15 ± 5.6	0.22
Tp-Te (msn)	96.85 ± 72	67.10 ± 10.51	0.01
Tp-Te/QT	0.22 ± 0.008	0.18 ± 0.02	0.01
Tp-Te/QTc	0.23 ± 0.01	0.16 ± 0.01	0.01

We evaluated cardiac functions using systolic and diastolic functions and coronary artery involvement with transthoracic echocardiography. These studies were performed by two echocardiographers using pulse-wave Doppler and M-mode echocardiography only due to the risk of infection. Five (7.1%) patients had pericardial effusion, and 20 (28.5%) patients had mild mitral insufficiency. Among these, only 5 had systolic dysfunction, with ejection fraction < 55%. The echocardiographic parameters of all patients are shown in Table 5. 

**Table 5:** Transtorasic echocardiographic parameters of the patients and control group.

Parameters	Patients(n = 70)	Controls(n = 70)	p
MV E (m/sec)	0.73 ± 0.05	0.78 ± 0.07	0.10
MV A (m/sec)	0.69 ± 0.08	0.64 ± 0.02	0.11
IVRT (msn)	58.1 ± 6.55	77.8 ± 7.35	<0.05
DT (msn)	94.4 ± 18.2	93.02 ± 4.5	0.77
IVSD (cm)	0.57 ± 0.07	0.60 ± 0.04	0.75
LVEDD (mm)	36.47 ± 6.22	36 ± 4.42	0.83
LVESD (mm)	28.29 ± 3.44	27.66 ± 3.22	0.69
LVPWD (cm)	0.70 ± 0.05	0.69 ± 0.04	0.89
FS (%)	29 ± 2.8	32 ± 2.4	0.92
EF(%)	61.2 ± 6.9	65 ± 3.8	0.78

E: early diastolic mitral inflow velocity, A: late diastolic mitral inflow velocity, IVRT: isovolumetric relaxation time, IVSD: interventricular septum diameter, LVPWD: left ventricle posterior wall diameter, LVESD: left ventricle end-systolic diameter, LVEDD: left ventricle end-diastolic diameter, FS: fractional shortening, EF: ejection fraction.

Three patients were diagnosed as having pediatric inflammatory multisystem syndrome (PIMS). These patients had coronary artery involvement with Z scores >2.5 (Table 6).

**Table 6:** Parameters of patients with paediatric multisystem ınflammatory syndrome.

Patient	Coronary involvement	Tr-T(ng/L)	NT-pro BNP(ng/L)	Tp-Te(msn)	Tp-Te/QT	Tp-Te/QTc
No. 1	LCA fusiform aneurysm	352	600	102	0.22	0.23
No. 2	LAD fusiform aneurysm	162	450	98	0.24	0.22
No. 3	LCA diffuse ectasia	66	400	92	0.22	0.23

LCA: Left coronary artery, LAD: Left anterior descending, NT-pro BNP: n terminal pro brain natriuretic peptide.

Table 7 demonstrates the regression analyses of the significant values, such as Tp-Te, Tp-Te/Qt, Tp-Te/Qtc, and IVRT, by means of troponin levels. In this table, Tp-Te/Qt is found to be a significant predictor of mortality and morbidity (p: 0 .031, OR: 1.01, 95% CI: 1.22–4.23)

**Table 7:** Regression analysis of the significant values.

Troponinlevels	B	S.E	Wald	p	Exp (B)	95% Confidenceinterval for Exp(B)	
						Lower bound	Upper bound
Tp-Te	0.060	0.061	0.94	0.33	1.06	0.94	1.19
Tp-Te/Qt	0.68	3.16	4.63	0.031	1.01	1.22	4.23
Tp-Te/Qtc	–5,18	26.10	0.039	0.84	0.006	3.46	9.75
IVRT	–0.023	0.05	0.20	0.65	0.97	0.88	1.07

Table 8 shows the univariate analyses of mortality and morbidity risk of these parameters by means of Covid-19 PCR positivity. Here, Tp-Te is an important predictor of mortality and morbidity (p: 0.02, OR: 0.99, 95% CI: 0.78–1.04).

**Table 8:** Univariate logistic regression prediction of mortality and morbidity.

Variable	c2	p	Odd ratio	95% CI
Age	34.07	0.51	0.99	0.62–0.93
Sex male	0.15	0.93	0.09	0,181–0.196
Tp-Te	40.06	0.02	0.99	0.78–1.04
Tp-Te/Qt	5.54	0.93	2.45	–10.13–12.21
Tp-Te/Qtc	6.49	0.16	3.7	–3.95–6.98
IVRT	3.52	0.85	0.02	–0.016–0.013

Dependent variable: Covid PCR positivity.

**Figure 3:**
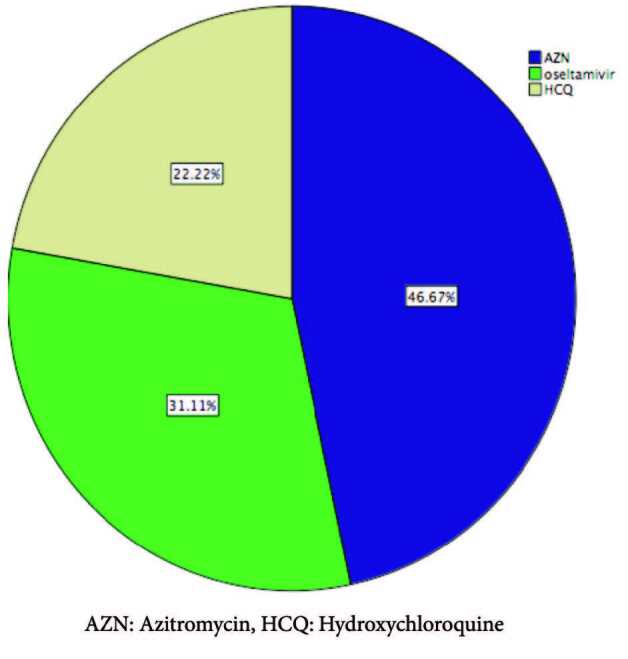
Treatment modalities of our patients.

Patients were treated according to Turkish Ministery of Health treatment protocol and nonspesific antimicrobial treatment (Table 9) (Figure 3) [14].

**Table 9:** Doses and administration methods of drugs to be used in childhood treatment.

Drug name		Daily dose and administration method on child patients	Duration of therapy (day)
First preference			
Hydroxychloroquine,200 mg tablet		First day 6.5 mg/kg/dose, 2 times a day hydroxychloroquine sulphate; maximumdose on first day: 400 mg/dose; continuing on days 2–5 at 3.25 mg/kg/dose,2 times a day hydroxychloroquine sulphate: maximum dose 200 mg/dose	5 days
Alternative treatment or in case of progress			
Lopinavir	250	Babies between 14 days - 6 months old:	10–14 days
mg/ritonavir	50 mg	Lopinavir component 16 mg/kg PO BID	
tablet		Children between 6 months - 18 years: 15–25 kg: 200–50 mg PO BID	
		26–35 kg: 300–75 mg PO BID	
		>35 kg: 400–100 mg PO BID	
Or in adolescents older than 15 years			
Favipiravir200 mg tablet		2 × 1600 mg loading,2 × 600 mg maintenance	5 days
Additional suggestions to antiviral treatment in confirmed COVID-19 patients who have been put into intensive care unit yet have organ dysfunction despite supportive therapy; please refer to the intensive care treatment in the guidelines for patients developing MAS or hemophagocytic syndrome.			

AZN: Azitromycin, HCQ: Hydroxychloroquine.

## 5. Discussion

Most studies suggest that the clinical manifestations of children are less severe than in adultsThe Republic of Turkey Ministery of Health COVID-19 Study of Scientific Board. https://hsgm.saglik.gov.tr/depo/birimler/goc_saglıgı/covid19/rehber/COVID-19_rehberi20200414_eng_v4_002_14.05.2020.pdf.Published2020. [Accessed 14th April 2020]. . Pediatric patients respond to SARS-CoV-2 exposure differently. Neonates, children, and adolescents aged <18 years with COVID-19 are less likely to have any symptoms. Questions remain, however. Despite the less severe illness, are there any clues of ECG or TTE parameters during the illness, and are children safe from the virus as is thought?

In patients, hypotension, arrhythmias, and sudden cardiac death (SCD) were described as possible SARS‐CoV manifestations [14]. In a cohort of 121 patients, Yu et al. demonstrated that sinus tachycardia was the most common cardiovascular finding with an overall incidence of 72%. The mean duration of persistent tachycardia was 12.7 days with a mean heart rate of 117 (range, 102–150) beats/min, and the tachycardia remained persistent in nearly 40% of patients within 30 days after hospital discharge. The incidence of tachycardia during the third week of hospitalization, when most patients were afebrile, could be related to drug treatment or increased sympathetic tonus [15].

In our study, we found that the mean heart rate was 120 (range, 105–164) beats/min at the beginning of the illness, and 112 beats/min after 1 week of treatment when the patients were afebrile. The mean heart rate after 1 month was 94 beats/min, which was still slightly increased. This can be a result of increased sympathetic tonus due to hypoxia and fever, even in children. Ece et al. also demostrated significantly elevated heart rate in COVID-19 patients on their recent report [16].

Arrhythmia has been reported as a significant issue in adult patients [17]. They may be secondary to myocardial injury, direct effects of cytokines on sodium and potassium channels [18], metabolic disturbance [19], hypoxia [20], and genetic dysrhythmias, which may be revealed by acute illness or significant stressors, such as Brugada syndrome [20] and long QT syndromes [21]. We observed no significant arrhythmias among our patients, but arrhythmias can be a great concern if they develop because they can cause serious complications. 

In the current study, 47.1% of patients had fQRS. fQRS can be defined as the presence of additional R’ waves or a notch in the nadir of the R or S wave (fragmentation) in two contiguous leads in a routine 12-lead ECG [23]. It is an important predictor of mortality and arrhythmic events in many cardiac diseases such as myocardial infarction, myocarditis, cardiomyopathies, valvular heart disease, congenital heart disease, and cardiac channelopathies [24–28]. In our study, 25 of 70 (35.7%) patients had fQRS without increased troponin levels. This can be explained because fQRS can be seen without myocarditis and can be an early significant ECG marker during the course of the disease. 

Despite the illness and some ECG changes, none of the patients in our study had either arrhythmia or pathologic ST-T changes. In a study by Samuel et al. [29], 17% of patients had significant arrhythmias; some of the arrhythmias resolved spontaneously, whereas others needed antiarrhythmic therapy. Wang et al. found arrhythmias at a rate of 16.7% in a group of adult patients [17].

Ho et al. reviewed a total of 31 studies on 51 patients were included; 12 cases were confirmed myocarditis while 39 had possible myocarditis. In this group two patients had normal ECG, while 7 cases had ECG features consistent with myocarditis. Two patients had ventricular tachycardia, one had nonspecific intraventricular conduction delay and multiple premature ventricular complexes and one had a low atrial ectopic rhythm. Three patients showed ST-segment changes including elevation and depression, two had T wave inversions, one had diffuse U waves and one had low-voltage QRS complexes in limb leads [30].

In our study, PR, QRS, QTc, QTd, Tp-Te, Tp-Te/QT, Tp-Te/QTc parameters were all significant compared with the control group. Despite of increased levels compared with the control group by means of QRS duration and QTc, they were still in the acceptable ranges. This can be explained as we immediately diagnosed and evaluate these children, therefore these can be accepted as the early results of ECG parameters. On the other hand, none of the patients had pathologic QTc prolongation during the illness or during its treatment. 

Thirty-six patients had PR prolongation, which can be a marker of myocarditis or subclinical myocarditis. Among these, only 25 had increased troponin levels. Increased ventricular repolarization dispersion (QTd) is associated with malignant arrhythmias and has prognostic importance in terms of mortality and sudden cardiac death [31]. However, it has lost its significance in repolarization, thus researchers have begun to search for another ECG parameter for ventricular repolarization [33,34]. During the last decades, the Tpeak-Tend interval has been intensively investigated as an ECG marker of arrhythmic and mortality outcomes in various disorders, including chronic and acute forms of ischemic heart disease. Tpeak-Tend demonstrated an association with malignant arrhythmias in such cases [34].

Tp-Te interval and Tp-Te/QT ratio have been evaluated as actual markers of increased dispersion of ventricular repolarization [35,36]. Prolongation of Tp-Te interval was related with increased mortality in Brugada syndrome, long QT syndrome, and in patients with acute myocardial infarction [36]. Tp-Te can be affected by body weight and heart rate, so it has some limitations for use as a repolarization marker. On the other hand, Tp-Te/QT and Tp-Te/QTc are not affected by heart rate or body weight. As a result, Tp-Te, Tp-Te/QT and Tp-Te/QTc are novel markers of ventricular repolarization. Recent studies showed that increased levels might be a marker of ventricular arrhythmias. Yayla et al. found increased levels of Tp-Te/QTc in a group of patients who underwent radiofrequency catheter ablation, those whose ejection fraction was less than 50% [37]. In our study, we observed significantly increased levels of Tp-Te, Tp-Te/QT, and Tp-Te/QTc compared with the control group. 

Gupta et al. claimed that the Tp-Te/QT ratio could be used as an index of arrhythmogenesis, even in the presence of short, normal or long QT intervals. Additionally, they indicated that the Tp-Te/QT ratio was a better marker of ventricular repolarization [36]. Ucar et al. found a prolongation in the Tp-Te interval, and an increase in the Tp-Te/QT and Tp-Te/QTc ratios in patients with acute myocarditis when compared with healthy subjects [38] In the present study, none of the patients had any arrhythmias such as ventricular premature contractions and ventricular tachycardia. This may be explained as subclinical altered properties of the myocardium causing electrophysiologic abnormalities represented by the Tp-Te and Tp-Te/QTc ratio, despite patients having a good clinical condition.

The best-known clinical condition in children regarding COVID-19 is PIMS (PIMS/Kawa-COVID-19). Pouletty et al. showed that severe disease and intensive care requirement was observed in 44% of their cases [39]. Gonzales et al. showed a relationship between COVID 19 and Kawasaki disease like symtoms in the meta analysis [40]. Loomba et al. also mentioned this relation in their review [41]. The characteristics of this syndrome resemble those of Kawasaki disease (KD), an inflammatory syndrome in children that can lead to coronary artery abnormalities due to vasculitis. Kawasaki disease may occasionally trigger macrophage activation syndrome (MAS), a condition in which there is uncontrolled activation and proliferation of macrophages and other cell types, and could lead to multiorgan system dysfunction. A relatively small number of children have developed PIMS related to the COVID-19 pandemic. PIMS has been characterized as the presentation of a child with high, persistent fever with elevated inflammatory markers such as neutrophilia, elevated C-reactive protein (CRP), and evidence of single or multiorgan dysfunction.

 In our group, during 3 months only 3 patients were diagnosed as having PIMS; they were treated with single-dose immunoglobulin (2 g/kg), and acetylsalicylic acid (first antiinflammatory after the antiaggregant dose). The patients had coronary artery dilatation (z score > 2.5), and elevated inflammatory markers. Only one patient needed intensive care unit treatment, because of the necessity of inotropic support and resolved the symptoms in a few days, as described in patient number two. 

In this study, we also evaluated the systolic and diastolic functions during the illness. On the TTE, 20 patients had mild mitral insufficiency, among whom only 5 had systolic dysfunction (ejection fraction < 55%). There was no significant difference between the patient and control groups, except for IVRT in terms of mean systolic and diastolic function parameters. IVRT was significantly lower than in the control group. As we all know, IVRT is a measure of time from the onset of the aortic valve closure spike artifact to the onset of the mitral valve opening spike artifact. The first cause of shortening may be the increased left ventricle pressure, despite of mitral valve insufficiency in this patient group. Another reason can be an occult atrial fibrillation ECG pattern that can be result as an elevated left atrial pressure. We think that, destruction and inflammatory process in the cellular levels may cause this and also diastolic dysfunction. 

## 6. Conclusion

Despite all the adult studies, the effects of COVID‐19 on myocardial function are not well established in children. The thought that children are less affected by the illness may be a misconception. We conclude that, physicians should be careful with the course of the disease even if the child is not very unwell, in terms of ECG parameters such as Tp-Te, Tp-Te/QT, Tp-Te/QTc, which are early markers of arrhythmia. Because of the consequences that can cause arrhythmia such as decreased IVRT, increased Tp-Te, Tp-Te/QT, and Tp-Te/QTc; acetyl salicylic acid can be used as co-treatment during treatment. Based on what has been discovered and hypothesized regarding cardiac involvement in COVID‐19, there is a need for further studies, especially in children.

## 7. Limitations

This study has several limitations. Firstly, we wanted to correlate the ECG parameters after the infection, but no patients wanted to come back to the hospital owing to the ongoing pandemic situation. Many evaluations on TTE could be performed, but these echocardiographic assessments were conducted very quickly with the desire to protect the echocardiographer from the virus.

## 8. Ethical standards

The study was approved by the local ethics committee of University (Date: 24.07.2020-09.2020.717), and the Turkish Ministry of Health (2020-06-08/T19-55-10).
